# Comparative and pangenomic analysis of the genus *Streptomyces*

**DOI:** 10.1038/s41598-022-21731-1

**Published:** 2022-11-07

**Authors:** Hiroshi Otani, Daniel W. Udwary, Nigel J. Mouncey

**Affiliations:** 1 DOE Joint Genome Institute, Lawrence Berkeley National Laboratory, Berkeley, CA 94720 USA; 2 Environmental Genomics and Systems Biology Division, Lawrence Berkeley National Laboratory, Berkeley, CA 94720 USA

**Keywords:** Bacterial genomics, Bacterial genes, Bacterial evolution, Industrial microbiology

## Abstract

Streptomycetes are highly metabolically gifted bacteria with the abilities to produce bioproducts that have profound economic and societal importance. These bioproducts are produced by metabolic pathways including those for the biosynthesis of secondary metabolites and catabolism of plant biomass constituents. Advancements in genome sequencing technologies have revealed a wealth of untapped metabolic potential from *Streptomyces* genomes. Here, we report the largest *Streptomyces* pangenome generated by using 205 complete genomes. Metabolic potentials of the pangenome and individual genomes were analyzed, revealing degrees of conservation of individual metabolic pathways and strains potentially suitable for metabolic engineering. Of them, *Streptomyces bingchenggensis* was identified as a potent degrader of plant biomass. Polyketide, non-ribosomal peptide, and gamma-butyrolactone biosynthetic enzymes are primarily strain specific while ectoine and some terpene biosynthetic pathways are highly conserved. A large number of transcription factors associated with secondary metabolism are strain-specific while those controlling basic biological processes are highly conserved. Although the majority of genes involved in morphological development are highly conserved, there are strain-specific varieties which may contribute to fine tuning the timing of cellular differentiation. Overall, these results provide insights into the metabolic potential, regulation and physiology of streptomycetes, which will facilitate further exploitation of these important bacteria.

## Introduction

Actinobacteriota, a group of gram-positive bacteria with high G + C content DNA, are one of the largest taxonomic units of bacteria, and are found in a variety of ecosystems^1^. They comprise numerous organisms relevant to human health as antibiotic and therapeutics producers and for biotechnological applications. The soil-dwelling genus *Streptomyces* is well-known for producing a variety of bioactive compounds as secondary metabolites^2^, with *Streptomyces coelicolor* A3(2) serving as a model organism for the genus due to a long history of extensive biological and chemical investigations and availability of its complete genome sequence for over 20 years. *Streptomyces* bioactive compounds include therapeutics such as streptomycin, the first remedy for tuberculosis, and avermectins, antiparasitic agents^3,4^. Other examples of industrial chemicals streptomycetes produce are antibiotics used in animal health, tylosin and monensin^5,6^. It is expected that there are thousands more waiting to be identified. In addition, several streptomycetes such as *Streptomyces viridosporus* are lignocellulolytic bacteria producing extracellular enzymes which modify lignocellulose, and catabolise carbohydrates and aromatic compounds derived from lignocellulose as carbon sources^7–9^. Lignocellulose is a constituent of plant cell wall and abundant and renewable feedstocks for chemical production such as biofuels and other bioproducts^10^. Because of the ability of streptomycetes to catabolise lignocellulose-derived compounds as carbon sources and produce a wide variety of chemical compounds as secondary metabolites, they may be ideal platforms for conversion of plant biomass into valuable chemicals. The other characteristic feature of the biology of streptomycetes is their complex lifecycle, forming branching vegetative hyphae and sporogenic aerial hyphae. This mycelial lifestyle is quite distinct from the majority of bacteria, resembling that of filamentous fungi. When spores encounter favourable conditions, they germinate, and grow to form hyphae, which extend to form vegetative mycelia and take up nutrients from surrounding substrates. In response to specific signals such as nutrient depletion, hydrophobic aerial hyphae extend into air, escaping from aqueous environments. Aerial hyphae synchronously divide by multiple septa and each compartment further develops and maturates forming a reproductive spore^11^. The regulation of this unique morphological development has been traditionally studied mainly in *Streptomyces coelicolor* A3(2), *Streptomyces griseus* NBRC 13350, and *Streptomyces venezuelae* NRRL B-65442, and a number of regulatory proteins controlling aerial mycelium formation and sporulation have been identified^12^. Many transcription factors controlling aerial mycelium formation and sporulation are termed Bld and Whi proteins, respectively. Of these, BldD and AdpA/BldH are global transcription factors, controlling genes not only involved in morphogenesis, but also in secondary metabolism^13–15^. With many of these regulatory proteins including BldD and AdpA/BldH conserved in these 3 species, morphological development is believed to be controlled by common regulatory cascades, with minor differences as some strain-specific regulatory proteins involved in this process have been recently discovered^12^. The mycelial lifecycle of streptomycetes results from extension of hyphal tips known as polar growth^16^. Cell wall materials such as peptidoglycans and other glycans are incorporated at the hyphal tips and their production and incorporation are controlled by multiple enzymes^17,18^. Of these enzymes, glucanase and lytic polysaccharide monooxygenase play crucial roles by controlling localised remodeling and degradation of peptidoglycan^19^.

Unlike morphological development, metabolic pathways involved in the biosynthesis of secondary metabolites and peripheral catabolic pathways are more diverse. A single *Streptomyces* genome, for example, generally encodes 25–50 detectable secondary metabolite biosynthetic pathways^20^. Because of the biotechnological potential of their metabolism, a number of *Streptomyces* genomes have been sequenced, which has revealed an ever-increasing number of unique metabolic pathways. This diversity of metabolic pathways is complemented by a large number of transcriptional factors encoded in the *Streptomyces* genomes, including transcriptional activators and repressors and sigma factors, which enable precise control of expression of specific metabolic pathways^21^. Acquisition of a number of genes involved in secondary metabolism, regulation, and morphological development resulted in expansion of the *Streptomyces* genome sizes, which typically range between 6 and 11 Mb^22^. Comparative genomic analyses of 4 and 17 *Streptomyces* genomes revealed that large fractions of their genomes were accessory and presumed to be dispensable^23,24^. These analyses also revealed that the majority of genes involved in secondary metabolism were parts of the accessory genomes and distributed in sub-telomeric regions of their linear chromosomes. A previous pangenome analysis of 122 *Streptomyces* genomes revealed that secondary metabolism and xenobiotic metabolism overrepresented the horizontally acquired genes, though gene acquisition through horizontal gene transfer was rare in the genus *Streptomyces*^25^. In addition to secondary metabolism, streptomycetes encode a number of enzymes which modify plant biomass, presumably to acquire them as carbon sources. Indeed, an analysis on microbiomes from lignocellulic biomass revealed that streptomycetes dominated these microbiomes and accounted for the largest fractions of glycoside hydrolases, a group of carbohydrate-active enzymes (CAZymes), suggesting that streptomycetes could be the major plant biomass degrading microbes^26^. Several streptomycetes are also associated with insects such as fungus-farming ants^27,28^. Nevertheless, the activity and versatility of streptomycetes to utilise carbohydrates, and their interaction with plants and other organisms are yet to be explored.

In this study, a comparative genomic analysis of 205 complete *Streptomyces* genomes was performed. This analysis revealed the diversity and commonality of their metabolism and regulation as well as cellular differentiation. This analysis also identified catabolically versatile strains, which may prove to be useful for conversion of biomass into industrial chemicals. Although there exist a few pangenomic analyses of streptomycetes, they focused on particular groups of genes such as lateral gene transfer and auxiliary genes^23–25^. Unfortunately, the data used in those studies are not available in public domains for immediate use. Therefore, researchers interested in using previously reported comparative genomics data for the purposes other than those described in those reports need to repeat the entire process. Instead, our data are publicly available with no restriction to use. Additionally, the analysis we conducted is the most comprehensive study of the *Streptomyces* pangenome using the greatest number of genomes and analysing a variety of metabolic and signalling pathways. Our data expand the understanding of the metabolism and physiology of streptomycetes and are expected to facilitate exploitation of the metabolic capabilities of these industrially useful bacteria.

## Results and discussion

### The *Streptomyces* genomes

**Figure 1:**
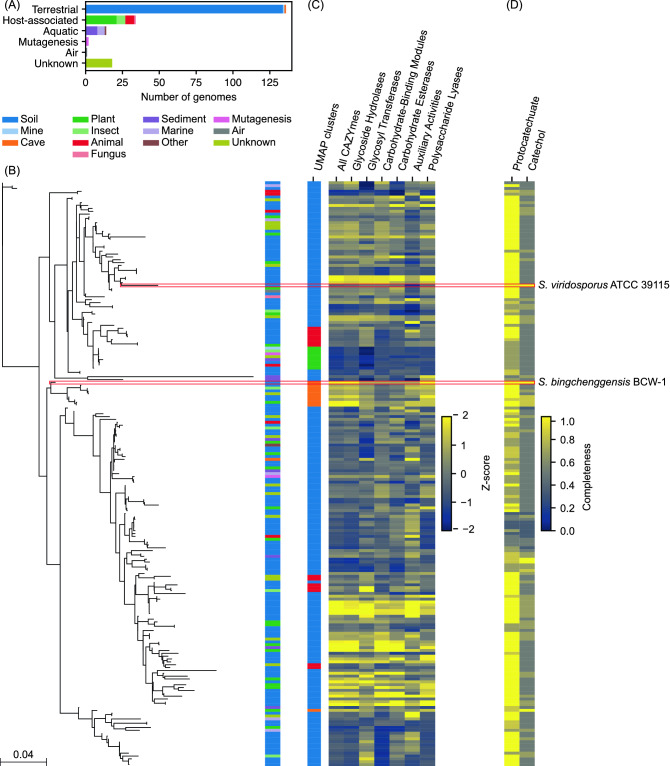
(**A**) Sources of the 205 streptomycetes used in this study. (**B**) Phylogenetic tree of the 205 streptomycetes using the 16S rRNA sequences. Sequences were aligned using PhyML. Red boxes indicate *S. bingchenggensis* BCW-1 and *S. viridosporus* ATCC 39115. (**C**) Clusters of the 205 strains based on the similarity of the 25 CAZymes families (see Fig. S3 for further information) and the relative numbers of CAZymes and CAZyme groups encoded in each genome. (**D**) Completeness of the protocatechuate and catechol catabolic pathways encoded in each genome. The pathway is complete if the genome encoded all the 6 enzymes each pathway requires. *S. bingchenggensis* BCW-1 and *S. viridosporus* ATCC 39115 encode the complete β-ketoadipate pathway responsible for protocatechuate and catechol catabolism. *S. bingchenggensis* BCW-1 also encodes a variety of CAZymes likely to be involved in polysaccharide depolymerization.

A total of 213 complete genome sequences of the genus *Streptomyces* were available at NCBI on 11 June 2020 (Table S1). Of these 213 genomes, 5 records were removed from NCBI later. Additionally, 3 genomes failed to satisfy the quality requirement for genome annotation by the Reference Sequence (RefSeq) project^29^. Therefore, these 8 genomes were removed from the further analyses. A total of 135 strains belonged to one of 91 species with *Streptomyces venezuelae* being most frequent (10 strains). At least 135 strains were isolated from soil samples (Fig. 1A). Of these, 9 strains were isolated from rhizosphere samples. Of the 34 strains that were isolated from another organism, 21 strains were isolated from plant samples. Other host-associated strains were isolated from animals, insects, and a fungus. Some of these strains may provide antimicrobial protection to their hosts^28^. Sources of 18 strains were not documented. There was no obvious relationship between the source of the strains and the similarity of the 16S rRNA sequences (Fig. 1B). *Streptomyces* sp. S1D4-11 possessed the largest chromosome with 12,276,515 nucleotides while *Streptomyces xiamenensis* MCCC 1A01550 possessed the smallest chromosome with 5,961,402 nucleotides. In order to ensure the consistency of gene predictions, annotations from the RefSeq project generated via the NCBI prokaryotic genome annotation pipeline were used throughout this study^30,31^. The number of protein-coding sequences (CDSs) varied between 5361 and 11,170.

### Pangenome analysis of streptomycetes

**Figure 2:**
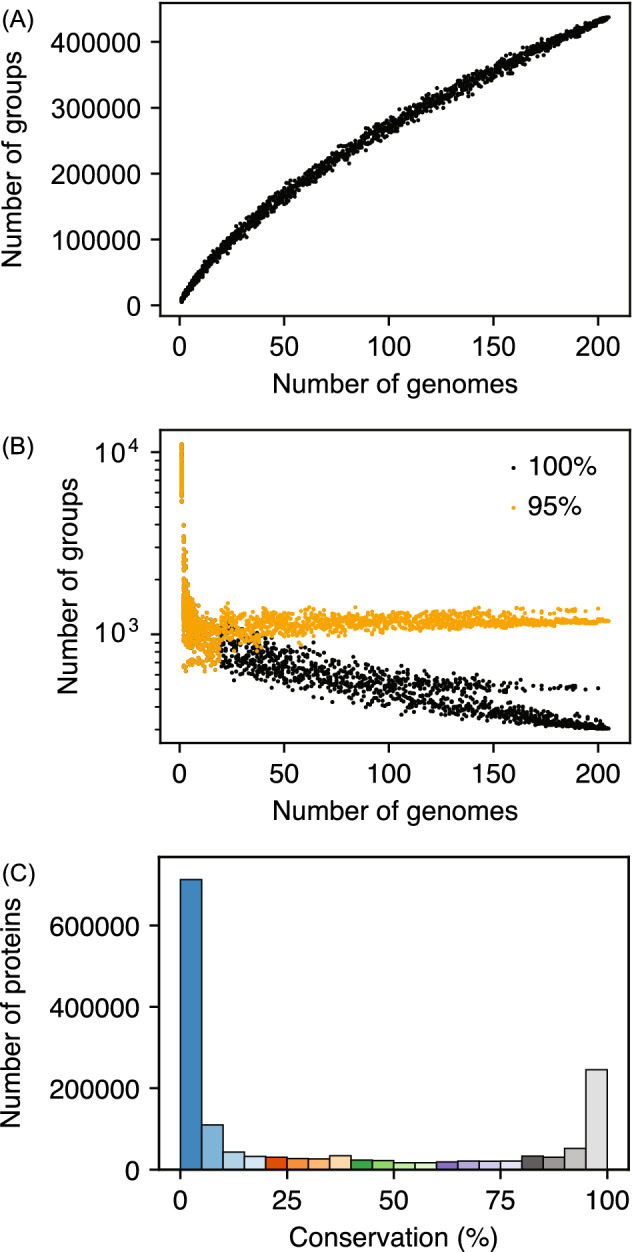
(**A**) Change in the pangenome size as a function of the number of genomes. X axis is the number of genomes used to construct the pangenome and Y axis is the number of orthologous groups identified in the same pangenome. (**B**) Change in the number of orthologous groups conserved in all the genomes and at least 95% genomes with the varying number of genomes. (**C**) The total number of proteins in each conservation bin from the *Streptomyces* pangenome. The bar colours correspond to the scheme indicated in Fig. 3A.

In order to identify genes with shared or strain-specific functions, a pangenome analysis of the 205 *Streptomyces* genomes was conducted. A protein pair with at least 80% amino acid sequence identity of the alignment covering at least 70% of both of the protein sequences were determined orthologues. Although more relaxed thresholds have been used in several studies such as 50% sequence identity and 50% sequence coverage^32,33^, such relaxed thresholds ended up clustering proteins known to exhibit distinct functions. For example, multiple sigma factors such as SigB, SigI, SigN and SigL encoded in *S. coelicolor* A3(2) were considered paralogues when the thresholds of 60% sequence identity and 60% sequence coverage were applied. While these sigma factor sequences are indeed relatively similar and they were presumed to recognise similar promoter sequences, their physiological roles are different^34–39^. Therefore, we decided to use more stringent thresholds to avoid potentially clustering proteins with relatively similar sequences and distinct functions. However, our stringent threshold may fail to cluster some orthologues when only partial conservation is sufficient such as active site residues for enzymatic activities as discussed below. The data files which include the source organism, predicted function and orthologous group assignment of each gene are available at the Secondary Metabolism Collaboratory (https://smc.jgi.lbl.gov/projects/). This analysis revealed 437,366 clusters of orthologous groups from 1,536,567 proteins. The total number of clusters increased as an additional genome was added (Fig. 2A). It indicates that streptomycetes possess an open pangenome and every strain is expected to encode a certain number of unique proteins. This openness of the pangenome suggests gene acquisition by lateral transfer and continuous gene sequence diversification in streptomycetes. Conversely, the number of orthologous groups present in all genomes decreased by an addition of a new genome and converged to 304 (Fig. 2B) consisting of 65,944 proteins (4.3%) when all 205 genomes were considered. Of these, 240 orthologous groups were present as a single copy in every genome. The number of core orthologous groups (conserved in at least 95% of genomes) converged to 1183 (Fig. 2B) consisting of 245,508 proteins (16.0%). Additionally, 289,378 proteins were unique and had no orthologues. *Streptomyces clavuligerus* F613-1 and *S. bingchenggensis* BCW-1 encoded the smallest (114) and largest (4691) number of strain-specific proteins, respectively. Nearly half of the proteins (712,556 proteins, 46.4%) were conserved in less than 5% of the genomes (10 genomes or less; Fig. 2C). While the number of core proteins (proteins conserved in more than 95% of strains) encoded in each genome was similar, the number of proteins conserved in less than 5% of strains encoded in each genome varied significantly (Fig. S1A). The latter number correlated well with the total number of proteins the corresponding genome encodes, suggesting that larger genomes tend to encode more unique proteins (Fig. S1B,C).

### Pathway analysis of the pangenome

**Figure 3:**
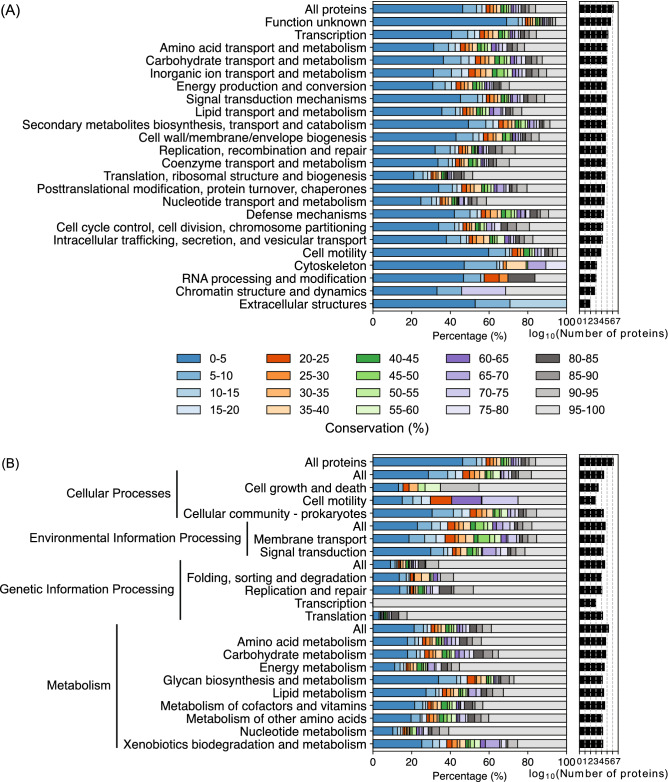
The total number of proteins in each COG (**A**) and KEGG (**B**) category and the percentage in each conservation bin.

Many of the orthologous groups conserved in all of the 205 strains had at least one protein that had already been characterised or was well annotated (proteins of which functions were not experimentally verified, but strongly predicted based on the homologies to characterised ones; e.g., ribosomal proteins). They included components of transcription and translation machinery, such as RNA polymerase and the ribosome. The largest cluster of orthologous groups consisted of 923 proteins, which were predicted to be cold-shock proteins^40^. *Streptomyces* sp. Mg1 encoded 12 homologues belonging to this cluster. Of 6 homologues encoded in *S. coelicolor* A3(2), SCO4505 and SCO0527 are abundantly produced under a non-stress condition^40^. Nevertheless, there were several orthologous groups conserved in all of the 205 strains that consisted of proteins without definitive functions. For example, the proteins in cluster 5 harboured a roadblock/LC7 domain, which is typically present in dynein proteins in eukaryotic organisms (cluster number assignments are included in the data files at the Secondary Metabolism Collaboratory). Such highly conserved uncharacterised orthologous groups are likely to encode important functions that have been overlooked. Functional categorization using the EggNOG database assigned 1,330,962 proteins in one of the COG categories and revealed the different degrees of conservation in each functional category^41^ (Fig. 3A). The largest number of proteins (509,538 proteins) were assigned to the “function unknown” category, of which the largest fraction (69%) belonged to orthologous groups encoded in 10 genomes or less (< 5%). The second largest functional category was “transcription” and consisted of 167,701 proteins. This included the large numbers of transcriptional activators, repressors, and sigma factors that *Streptomyces* genomes encoded, presumably to enable adaptation to the diverse and ever-changing environments that streptomycetes inhabit. Several categories had relatively higher proportions of core proteins. For example, 48.5% and 41.4% of proteins assigned to the “translation, ribosomal structure, and biogenesis” and “nucleotide transport and metabolism” were core, suggesting the important conserved roles that proteins in these categories play.

Next, we conducted a pathway analysis of the pangenome using the KEGG database^42^ and assigned 634,155 proteins to KEGG pathways (Fig. 3B). This analysis revealed the high degree of conservation of proteins belonging to “genetic information processing”. Most notably, all the proteins in the “transcription” category and more than 80% of proteins in the “translation” category were core proteins. The KEGG pathway analysis also revealed that streptomycetes dedicated 35% of proteins that were assigned to the KEGG pathways to the category “metabolism” (this category used in this study only includes metabolic pathways of primary metabolism and those of secondary metabolism are excluded due to their incompleteness in the KEGG database). Nearly 40% of proteins belonging to “metabolism” were highly conserved compared to the pangenome, of which 15% were highly conserved, corresponding to the fact that *Streptomyces* genomes encode several shared metabolic pathways, such as the TCA cycle and nucleotide biogenesis. The “xenobiotic biodegradation and metabolism” category had the smallest percentage (25%) of core proteins followed by the “glycan biosynthesis and metabolism” (27%) and “lipid metabolism” (33%) categories while more than 20% of proteins in these categories were conserved in less than 5% of the strains. This suggests that different strains potentially catabolise different organic compounds as carbon sources and produce structurally diverse peptidoglycans and lipids.

### Detailed analyses of important biological processes in streptomycetes

#### Catabolism of lignocellulose and aromatic compounds

**Figure 4:**
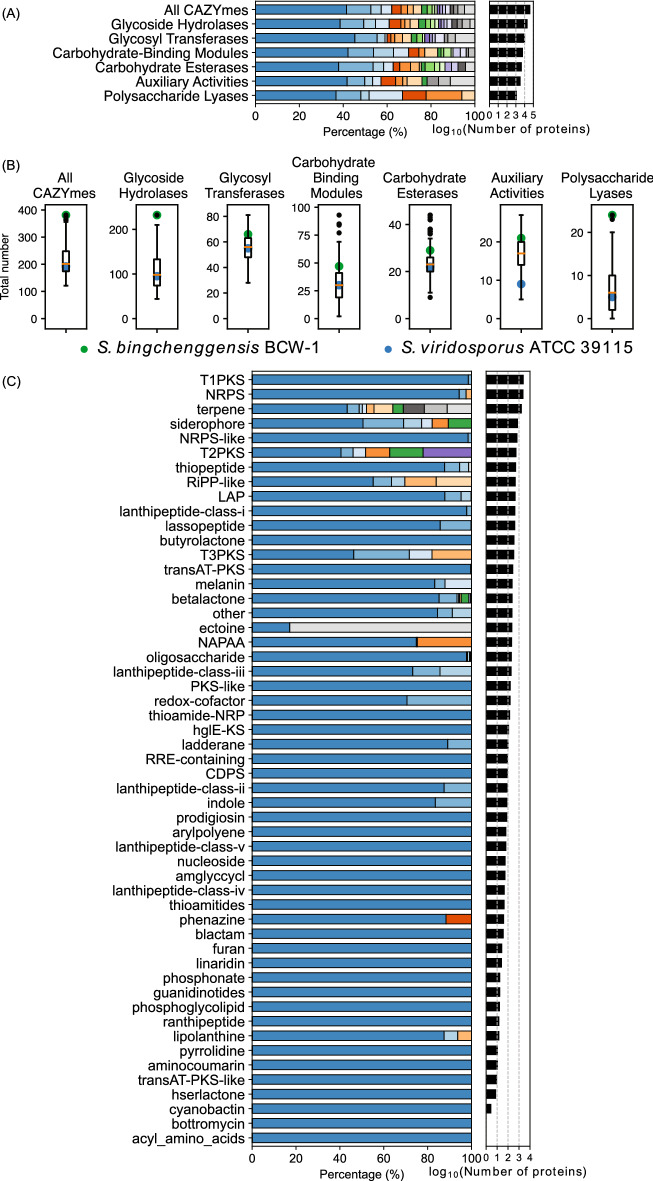
The total number of proteins in each CAZyme group (**A**) and antiSMASH BGC type (**C**) and the percentage in each conservation bin. (**B**) The total number of CAZymes and CAZyme groups encoded in each genome. The colour codes are the same as those in Fig. 3.

Actinobacteria contribute to global carbon cycling by breaking down plant biomass constituents, such as lignocellulose^43^. Several streptomycetes linked to the plant biomass degradation process have been isolated and studied^7^. They encode a large number of CAZymes, some of which are presumed to be responsible for decomposition of lignocellulose, especially cellulose. The *Streptomyces* pangenome encoded 44,504 proteins predicted to possess at least one protein domain representative to CAZymes (Fig. 4A; Table S2). *Streptomyces bingchenggensis* BCW-1 encoded the greatest number (381 proteins) of CAZymes while *Streptomyces spongiicola* HNM0071 encoded the smallest number (121 proteins). About 40% of the CAZymes were conserved in 10 strains or less (< 5%) while 2096 proteins (4.7%) were core. Interestingly, the proportions of CAZymes conserved between 5 and 55% of the strains substantially increased and the proportion of CAZymes conserved in less than 5% of the strains was smaller compared to the pangenome (Fig. S2A), suggesting that many carbohydrate modifying reactions could be shared by multiple, but only taxonomically close, organisms. Indeed, the numbers of CAZymes encoded in the phylogenetically related genomes were similar (Fig. 1B). Hence, we further analysed CAZyme families primarily conserved in 5–55% of the strains. We used 25 CAZyme families of which at least 70% of the members were conserved between 5 and 55% of the strains. A presence-absence matrix of the orthologous groups belonging to these CAZyme families was used to cluster the 205 strains based on the similarity of the presence/absence of each orthologous group. The Uniform Manifold Approximation and Projection for Dimension Reduction (UMAP) and Density-Based Spatial Clustering of Applications (DBSCAN) algorithms were used for this clustering analysis (Figs. 1C, S3A,B)^44,45^. The largest cluster contained 173 genomes. Interestingly, these 173 genomes spanned multiple taxonomic clades. Many other clusters were primarily restricted to taxonomically-related genomes. It is plausible that the CAZymes encoded in these 173 genomes, the largest cluster of the UMAP plot, were canonical *Streptomyces* enzymes which were lost or evolved in some taxonomic clades and the 25 CAZyme families on average evolved earlier than the rest of the pangenome (Fig. S3B).

Of the 6 CAZyme classes, the glycoside hydrolases (GHs) were most abundant (21,943 proteins) followed by glycosyl transferases (GTs; 11,366 proteins) (Fig. 4A). Polysaccharide lyases (PLs) were the smallest group with the greatest coefficient of variance (Fig. 4B; Table S2). No PL was conserved in more than 79 strains (38.5%). Interestingly, the number of GHs in a given genome correlated well with the numbers of PLs, carbohydrate-binding modules (CBMs) and carbohydrate esterases (CEs) while correlation with the numbers of GTs and auxiliary activities (AAs) were relatively poor (Fig. S2B). GHs, CEs and PLs catalyse cleavage of glycosidic bonds by hydrolysis, esters by hydrolysis, and glycosidic bonds by β-elimination, respectively, and are generally involved in depolymerisation of polysaccharides, while GTs catalyse glycosidic bond formation. Therefore, it is plausible that strains that encode greater numbers of GHs, CEs and PLs are capable of depolymerising a wide variety of polysaccharides. *S. bingchenggensis* BCW-1 encoded the greatest number of GHs and PLs while *Streptomyces chartreusis* NRRL3882 encoded the greatest number of CEs (Fig. 4B).

The KEGG pathway analysis revealed that each *Streptomyces* strain encoded between 47 and 170 proteins (97 proteins on average) predicted to be involved in “xenobiotic biodegradation and metabolism”. Many metabolic pathways in this category are involved in degradation or catabolism of aromatic compounds, suggesting the potential of streptomycetes to degrade a variety of aromatic compounds in natural environments. Indeed, many Actinobacteria including streptomycetes are known to catabolise many aromatic compounds including benzoate and cinnamate derivatives as carbon sources^9,46,47^. Aromatic compounds are constituents of lignin, and therefore components of lignocellulose. Many aromatic compounds are converted to catechol or protocatechuate, which are further catabolised via the β-ketoadipate pathway to acetyl-CoA and succinyl-CoA^9,48^. As these molecules are precursors of high value chemicals, such as polyketides and triacylglycerols, conversion of the lignin-derived aromatic compounds into acyl-CoA via the β-ketoadipate pathway is an important step for the production of such bioproducts from plant biomass^49,50^. The catechol and protocatechuate catabolic pathways consist of 6 biochemical reactions, of which the last 3 reactions are shared by both pathways. Of the 205 strains, 114 strains were predicted to encode the complete protocatechuate catabolic pathway (Fig. 1D). However, only 5 strains were predicted to encode the complete catechol catabolic pathway (Fig. 1D). Only *S. bingchenggensis* BCW-1 and *Streptomyces viridosporus* ATCC 39115 were predicted to encode both of the protocatechuate and catechol catabolic pathways completely. *S. bingchenggensis* BCW-1 also encoded the greatest number of CAZymes of all the 205 strains while the number of CAZymes *Streptomyces viridosporus* ATCC 39115 encoded was relatively small (Figs. 1D and 4B). Therefore, we expect that *S. bingchenggensis* BCW-1 is capable of catabolising a wide range of plant biomass constituents, though this has not been previously reported or confirmed. Coincidentally, *S. bingchenggensis* BCW-1 encodes the greatest number of strain-specific proteins, supporting its unique catabolic capabilities.

#### Secondary metabolism

In addition to catabolising a variety of organic compounds as carbon sources and producing essential biomolecules such as nucleotides and lipids as primary metabolites, streptomycetes produce a wide variety of nonessential chemical compounds as secondary metabolites^51^. A secondary metabolite is typically biosynthesised by a series of biochemical reactions catalyzed by dedicated enzymes. Often, all the enzymes involved in the biosynthesis of the same secondary metabolite are encoded in the same genomic locus, forming a biosynthetic gene cluster (BGC). Using antiSMASH v6.0.1^52^, BGCs present in each genome were predicted (Table S3). The total number of BGCs ranged from 18 (*Streptomyces* sp. CLI2905 and *Streptomyces tendae* 139) to 53 (*Streptomyces hygroscopicus* XM201 and *Streptomyces* sp. YIM 121038) with the mean and median numbers of 31.8 and 31, respectively. Of the 70 BGC types that antiSMASH v6.0.1 supports, the largest BGC type (1–20 BGCs per genome, a total of 1353 BGCs) was non-ribosomal peptide synthases (NRPSs) followed by terpenes (3–12 BGCs per genome, a total of 1254 BGCs) and type 1 polyketide synthetases (T1PKS; 0–19 BGCs per genome, a total of 1079 BGCs) (Fig. 4C, Table S4). Each BGC comprises at least one core enzyme responsible for the formation of core structures of secondary metabolites. Our pangenome analysis revealed that almost 80% of the core enzymes were conserved in less than 5% strains (Fig. 4C). The notable exception was the ectoine BGC type, presumably because of the important function of ectoine, the canonical member of this BGC type, as a protectant from salt and heat stresses^53^. Terpene BGC had 3 orthologous groups conserved in at least 80% strains, all of which were encoded in the BGC for hopanoids, one of the constituents of bacterial membrane lipids^54^. While *S. coelicolor* A3(2) wild-type produced hopanoids, the mutants unable to form aerial mycelia did not detectably produce them^55^. This suggests that the majority of streptomycetes produce structurally similar hopanoids, presumably as a constituent of membranes in aerial mycelia. Interestingly, geosmin cyclase, GeoA, from *S. coelicolor* A3(2) was conserved in only 79 genomes. Though geosmin cyclase was bioinformatically identified in a number of actinomycetes including streptomycetes, the homology of the overall sequences varies while the active site motifs are more highly conserved^56^. Indeed, 3 out of 4 active sites of GeoA in *S. coelicolor* A3(2) and its closest homologue in *S. venezuelae* NRRL B-65442 were identical and the other active site had a single residue substitution while their overall identity was below the threshold used in this study. For such enzymes, it may be more appropriate to consider conservation of active sites and residues involved in substrate selection rather than an overall sequence alignment.

Several BGC types consisted of core enzymes conserved in less than 5% strains. Of them, the BGC type butyrolactone had the proportion (100%, 386 proteins) of core enzymes conserved in less than 5% strains. These enzymes are responsible for transfer of the acyl moiety of β-ketoacyl-CoA to dihydroacetone phosphate, which is followed by spontaneous or enzymatical conversion to gamma-butyrolactones (GBLs), such as A-factor, by intramolecular aldol condensation, dephosphorylation, and reduction^57^. Several GBLs serve as signalling molecules for inter- and intraspecies cell–cell communications^58^. The antiSMASH analysis revealed that 177 of the 205 strains used in this study (86%) were predicted to encode at least one core enzyme for GBL biosynthesis. Of the 386 core enzymes for GBL biosynthesis, 171 had no orthologues and were, therefore, encoded in single strains (Fig. S4A), which suggests that many organisms produce strain-specific GBLs. Using an organism-specific chemical compound as a signalling molecule is one of the easiest ways to detect cells from the same organism and sense the population and our data suggest that the majority of streptomycetes potentially use GBLs for intraspecies communication.

NRPSs and PKSs were the most abundant core enzymes that antiSMASH identified. These enzymes are activated by incorporation of the phosphopantetheinyl side chain of coenzyme A into their peptidyl carrier domain or acyl carrier domain, which is catalysed by phosphopantetheinyl transferases (PPTases)^59^. A total of 990 PPTases were identified from the pangenome and each genome encoded 1–12 PPTases with median value of 4 (Fig. S4B). *S. collinus* Tu 365 encoded 12 PPTases. The pangenome analysis revealed 448 orthologous groups which PPTases were clustered into. Of the 448 orthologous groups, the largest group was conserved in 203 strains (Fig. S4C). One of the proteins in this largest orthologous group, AcpS in *S. coelicolor* A3(2), is a promiscuous enzyme which activates multiple ACPs including a fatty acid synthase ACP and actinorhodin synthase ACP, and is presumed to be a housekeeping PPTase as deletion of *acpS* was unsuccessful^59^. The conservation of AcpS in 203 strains suggests that they use AcpS to activate biosynthesis of housekeeping fatty acids and some polyketides and non-ribosomal peptides. Prokaryotic PPTases are classified into 2 types, AcpS- and Sfp-types^59^. AcpS-type PPTases are small and possess only a single 4’-phosphopantetheinyl transferase domain. Sfp-type PPTases are larger and possess a second 4’-phosphopantetheinyl transferase domain. The majority (901) of the PPTases the *Streptomyces* pangenome encoded were AcpS-types. *S. collinus* Tu 365 encoded 12 AcpS-type PPTases, and *S. venezuelae* strains NRRL B-65442, ATCC 21113, and ATCC 10712 and *Streptomyces* sp. DSM 40868 encoded 11 AcpS-type PPTases. Importantly, 9 PPTases *Streptomyces* sp. DSM 40868 encoded were unique. *S. venezuelae* strains, NRRL B-65442, ATCC 21113 and ATCC 10712 shared 1 PPTase unique to them and 7 PPTases that were conserved in them and *S. venezuelae* ATCC 10595, which encoded 10 PPTases. Although there were only 89 Sfp-type PPTases that the *Streptomyces* pangenome encoded, their pattern of distribution was distinct from that of the AcpS-type PPTases (Fig. S5). *Streptomyces hundungensis* BH38 encoded 3 Sfp-type PPTases, of which 2 were unique. However, this strain encoded only 4 AcpS-type PPTases. *Streptomyces albireticuli* MDJK11 encoded 2 Sfp-type PPTases that were unique to this strain in addition to 6 AcpS-type PPTases, of which 5 were unique. Interestingly, *Streptomyces aureoverticillatus* HN6 encoded 2 unique Sfp-type PPTases and 6 AcpS-type PPTases with varying degrees of conservation. While several AcpS-type PPTases are known to activate acyl-carrier proteins of fatty acid and polyketide synthetases, canonical members of Sfp-type PPTases modify carrier proteins of PKSs and NRPSs. Substrate specificities of PPTases vary. Hence, strains encoding a diverse set of PPTases are likely to be capable of activating a wide variety of PKSs and NRPSs and could be desirable for heterologous expression of PKS and NRPS BGCs.

#### Sigma factors and transcription factors

**Figure 5:**
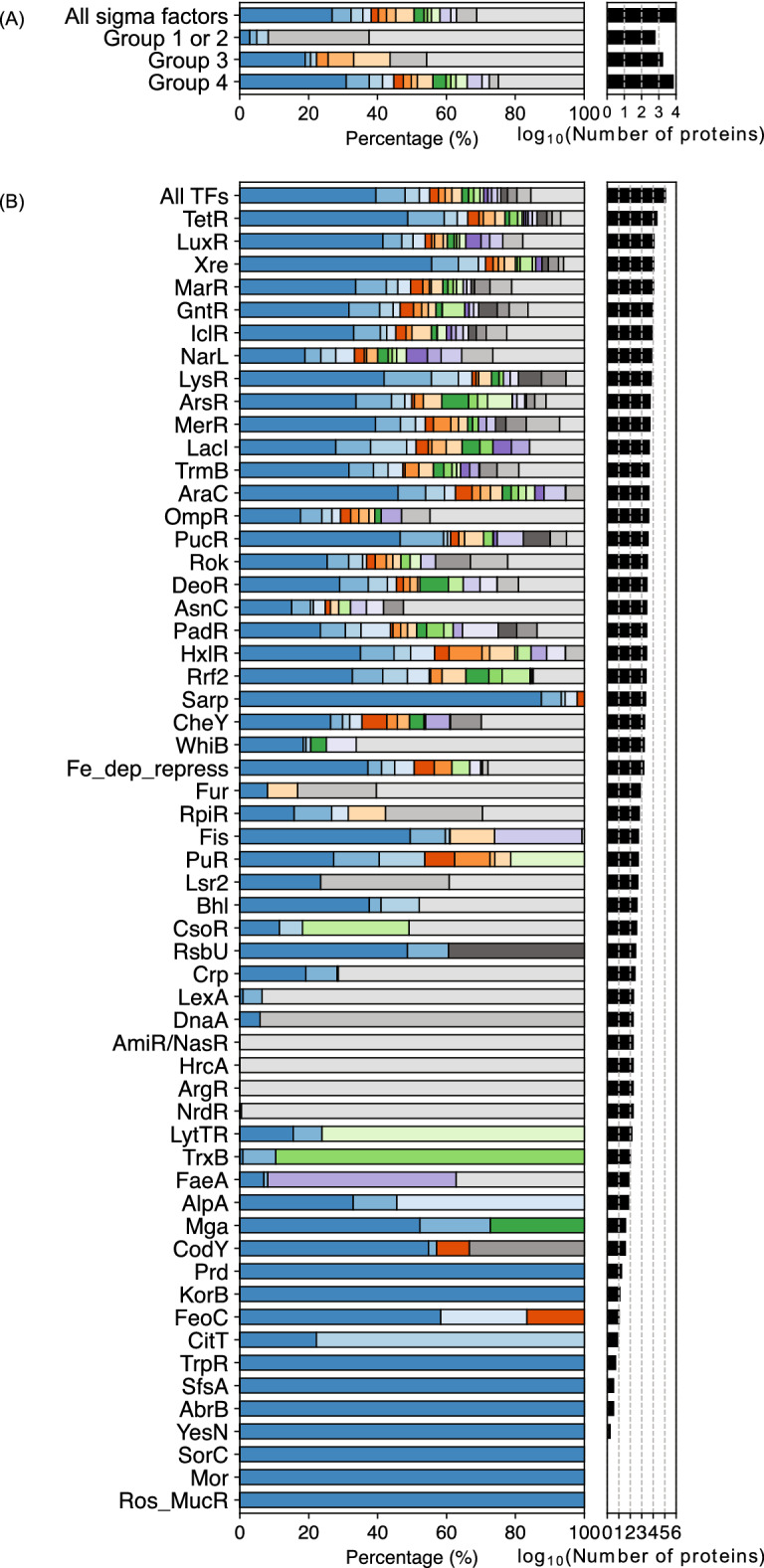
The total number of proteins in each sigma factor group (**A**) and other transcription factor family (**B**) and the percentage in each conservation bin. The colour codes are the same as those in Fig. 3.

Precise control of gene expression is a key to modulating cellular metabolism and physiology, and adapting to ever-changing environments. Bacteria use sigma factors and other transcription factors such as transcriptional activators and repressors to control gene expression, primarily initiation of transcription. Sigma factors are dissociable components of RNA polymerase and are essential for promoter recognition and transcription initiation^60^. Many bacteria encode multiple sigma factors, each of which recognises a specific set of its own target promoters and allows expression of condition-specific genes. Sigma factors comprise 2 protein families, σ^70^ and σ^54^, although the σ^70^ family is larger and no σ^54^-family sigma factors have been reported in streptomycetes to date. Consistent with this, the Pfam domain search only identified σ^70^-family sigma factors in the 205 *Streptomyces* genomes. A total of 9741 sigma factors were identified, and the number of sigma factors per genome varied from 27 (*Streptomyces luteoverticillatus* CGMCC 15060 and *Streptomyces* sp. NHF165) to 80 (*Streptomyces* sp. RLB3-17) with a median value of 46. The σ^70^-family sigma factors are categorised into 4 groups based on domain organisation and function^60,61^. All of the 9741 sigma factors were categorised into 3 groups (group 1 or 2, group 3, or group 4; group 1 and 2 sigma factors consist of the same minimum set of domains and may not be distinguished unambiguously without functional characterisation) (Fig. 5A). More than 60% of sigma factors belonging to the group 1 or 2 were core proteins. As the group 1 sigma factors (σ^HrdB^) are involved in expression of essential housekeeping genes^62–66^ and some group 2 sigma factors are presumed to be involved in general stress response^37,67–69^, many streptomycetes use these sigma factors to express genes involved in the basic biological processes. The largest number of sigma factors (75%) belong to group 4 (or extracytoplasmic function subfamily) and they were the least conserved (Fig. 5A). The canonical members of group 4 control expression of genes with specialised function such as response to specific stress conditions^60,70^. We expect strain-specific sigma factors to control strain-specific genes, such as peripheral metabolic pathways and adaptation to specific environmental conditions. For example, of all the characterised sigma factors in streptomycetes, the least conserved was Orf21 in *Streptomyces clavuligerus* ATCC 27064 (NRRL 3585 in the original report), which is suggested to be involved in clavulanic acid production^71^, and was conserved only in *S. clavuligerus* ATCC 27064, *S. clavuligerus* F613-1 and *S. clavuligerus* F1D-5. Highly conserved sigma factors, on the other hand, are likely to be involved in fundamental biological processes. Of the 15 core orthologous groups, 13 groups had at least one member which had been characterised previously with the functions of 11 members known (Table S5). These groups included σ^R^, which responds to thiol-perturbing signals^72,73^, σ^BldN/AdsA^, σ^WhiG^ and σ^F^, which control the onset of aerial mycelium and spore formation and maturation^74–77^, and σ^HrdB^ and σ^ShbA^, which control the expression of the housekeeping genes and the principal sigma factor gene, respectively^62,64,66^. It is, therefore, plausible that the uncharacterised highly conserved orthologous groups, 588 and 1166, might play important roles. Several previously reported transcriptomics data show that genes encoding the members of the orthologous group 588, *SCO5147* in *S. coelicolor* A3(2) and *vnz_RS23885* in *S. venezuelae* NRRL B-65442, are highly transcribed in later growth phases^78–80^, suggesting their possible role in stationary phase and its investigation is warranted.

Members of other types of transcription factors such as transcriptional activators and repressors, instead of forming a complex with the RNA polymerase core enzyme as a subunit, bind to specific DNA sequences around promoter regions and activate or repress transcription of certain genes. These types of transcription factors are classified into different protein families depending on domain architectures^81^. Using the domain architectures described by Ortet *et a*., 2012^81^, 130,380 transcriptional activators and repressors were identified and classified (Fig. 5B). We found that the number each genome encoded in streptomycetes ranged from 401 to 1,018 with the median value of 612. Nearly 40% of transcription factors (52,651) were conserved in 10 strains or less (less than 5%). Of these, 15,018 transcription factors were strain-specific, suggesting the existence of a large number of species or strain-specific transcriptional regulatory systems. The TetR family was the largest transcription factor family with 23,581 members (57–199 per genome). Of the transcriptionfactor families that had at least 205 members, the SARP (*Streptomyces* antibiotic regulatory protein) family was least conserved (Fig. 5B). Many SARP family regulators were found to directly control secondary metabolite production in streptomycetes and are frequently encoded within secondary metabolite BGCs^82^. These cluster-situated regulators are typically responsible for controlling the expression of their own BGCs. Because many secondary metabolite BGCs were strain-specific (Fig. 4), it is expected that transcriptional regulation of the majority of BGCs should be BGC-specific. Indeed, we found that transcription factors and sigma factors encoded inside secondary metabolite BGCs were less conserved (Fig. S6). While species- and strain-specific regulators could be controlling expression of unique genes, highly conserved regulators are likely to regulate conserved genes and be involved in more conserved transcriptional regulatory mechanisms. Of the 98 orthologous groups that were core (Table S6), 55 groups had at least one member which had been functionally characterised previously. These groups included the cyclic AMP receptor protein Crp^83^, BldC, BldD, BldM, WhiB, WhiD, WhiH, and WhiI, involved in morphological development^78,84–87^, and CseB, LexA, OsaAB and PhoP involved in stress responses and homeostasis^88–91^. Similar to sigma factors, some uncharacterised transcriptional activators and repressors that are core may play fundamental roles in *Streptomyces* biology.

#### Morphogenesis

**Figure 6:**
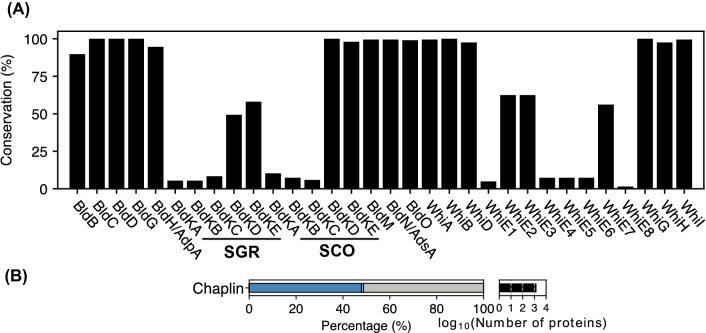
(**A**) Conservation of Bld and Whi proteins. (**B**) The total number of proteins in chaplin family and the percentage in each conservation bin. The colour codes are the same as those in Fig. 3.

The pangenome analysis revealed that several genes involved in aerial mycelium formation and sporulation are highly conserved (Fig. 6A), suggesting that the regulation of aerial mycelium formation and sporulation is a conserved process. However, many BldK and WhiE proteins are conserved in a relatively small number of strains. The oligopeptide transporter, BldK, originally identified in *S. coelicolor* A3(2) is composed of 5 proteins (BldKA-BldKE) and is responsible for uptake of a 655 Da peptide, which is presumed to act as a molecule signalling initiation of aerial mycelium formation^92,93^. Its functional orthologue in *S. griseus* NBRC 13350 exhibits relatively low sequence similarities (20–59%)^94^. Indeed, we found that BldKA (permease), BldKB (ABC transporter substrate-binding protein) and BldKC (permease) in *S. coelicolor* A3(2) and *S. griseus* NBRC 13350 were only conserved in 5–10% of the strains, while BldKD and BldKE (ABC transporter ATPases) in *S. coelicolor* A3(2) and in *S. griseus* NBRC 13350 were more highly conserved (Fig. 6A). This low-level conservation of the substrate-binding protein suggests that different strains may use different peptides as signalling molecules for aerial mycelium formation. WhiE proteins are responsible for production of gray-pigmented aromatic polyketides in *S. coelicolor* A3(2)^95^. The low-level conservation of the WhiE proteins suggests that the aromatic polyketides that the *whiE* genes encode are presumably produced by only limited strains. Indeed, different spore pigments have been observed such as green, blue, and red, in different strains^96^.

While the Bld and Whi proteins contribute to determining the timing of aerial mycelium formation and sporulation, several other proteins are involved in formation of aerial mycelia and spore chains. A specific group of amyloid proteins in streptomycetes, known as chaplins, are essential components of the hydrophobic coat of aerial mycelia and spores^97^. A total of 1500 proteins were predicted to possess this domain from the *Streptomyces* pangenome (Fig. 6B). *Streptomyces tsukubaensis* AT3 encoded the greatest number (13) of chaplins and 4 strains encoded a single chaplin. Interestingly, 768 chaplins (51%) belonged to the same orthologous group and were conserved in 193 strains. In *S. coelicolor* A3(2), 8 chaplins are encoded and ChpE and ChpH are the minimally required chaplins for aerial mycelium formation while ChpC is required for formation of robust, sporulating aerial mycelia^98^. Of these proteins, ChpE and ChpH belonged to the orthologous group conserved in193 strains while ChpC was conserved in only 14 strains. This suggests that the majority of streptomycetes use ChpE and ChpH homologues to form basal aerial mycelia, and more unique chaplins are involved in formation of robust mycelia, presumably in a strain-specific manner.

## Conclusion

Actinobacteria, especially streptomycetes, are known for their metabolic prowess with huge potential for biotechnological applications in the production of secondary metabolites and catabolism of lignocellulose constituents. Streptomycetes inhabit diverse environmental conditions and have acquired a large number of metabolic and regulatory genes, presumably as a result of adaptation. Our pangenome analysis shows that many streptomycetes encode a large number of unique enzymes involved in secondary metabolite biosynthesis and catabolism of carbohydrates and aromatics. Of the 205 strains used in this study, *S. bingchenggensis* BCW-1 encodes the greatest number of enzymes involved in lignocellulose catabolism. Carbohydrate-active enzymes underwent evolution earlier than the majority of other genes, resulting in a large number of enzymes conserved in taxonomically-related genomes. The exact mechanism of how this evolution occurred is still unknown, which merits further investigation. The *Streptomyces* pangenome also encodes unique gamma-butyrolactones, presumably as species- or strain-specific signalling molecules, while ectoine and hopanoid products are likely structurally similar. The majority of highly conserved regulatory proteins, including transcriptional activators, transcriptional repressors and sigma factors, appear to play important roles in the *Streptomyces* physiology. However, the majority of regulatory proteins encoded inside secondary metabolites BGCs are poorly characterised and it is important to elucidate their functions to facilitate the discovery of new secondary metabolites. Although the majority of genes involved in morphological development and the metabolism of second messengers, which control the timing of morphological development, are highly conserved, strain-specific transporters and second messenger metabolic enzymes fine-tune the timing of development, presumably in a strain-specific manner. One limitation of this study is that we used the identity of the sequence alignment and the coverage of the alignment as the criteria when determining orthologues. However, the conservation of the active sites and other residues involved in substrate recognition is more important than homology of the entire sequences for certain enzymes as evident with geosmin cyclase. For such enzymes, taking account for active site motifs if known may be more suitable. Nevertheless, our dataset revealed genetic potential of a number of streptomycetes, which may be further characterised for secondary metabolite discovery and production, and biomass utilisation. In addition to the analyses presented here, we view this dataset to be a community resource to be probed in many different ways and so we have made our dataset freely and openly available in JGI’s Secondary Metabolism Collaboratory (https://smc.jgi.lbl.gov/projects/). Though this study used the 205 complete *Streptomyces* genomes, which is the largest scale of the comparative genomic analysis of streptomycetes, this number is still less than 1% of the > 20,000-member genus. In order to harness the complete metabolic capability of this biotechnologically important genus, it is warranted to sequence and explore a much larger number of their complete genomes.

## Methods

### Genome analysis

A total of 213 complete *Streptomyces* genomes were retrieved from the NCBI Reference Sequence Database (RefSeq) on 11 June 2020^30^. Phylogenetic analysis using the 16S rRNA sequences was performed using PhyML 3.0^99^. BLAST + v2.10.1^100^ was used to cluster proteins using the following criteria: E-value < 10^–5^, identity ≥ 80%, coverage ≥ 70%. Clusters (or orthologous groups) were identified using the Python package, NetworkX^101^.

### Functional annotation and prediction

A COG term was assigned to each protein using the EggNOG database v5.0 and HMMER v3.1^41,102^. The independent E-value threshold of 10^–10^ was used. KofamKOALA v1.3.0 was used to assign KO terms using the independent E-value threshold of 10^–10^ and the KO term specific score threshold^103^. KEGG pathways present only in eukaryotes or in the “biosynthesis of secondary metabolites” category were excluded from the analysis. AntiSMASH v6.0.1 was used to predict secondary metabolite biosynthetic gene clusters^52^. Carbohydrate active enzymes were predicted using dbCAN2 v9^104^. The Pfam protein families database v33.1 and the SMART protein domain annotation resource v8 were used to assign protein domains using the independent E-value threshold of 10^–1^^105,106^. The Pfam domain, “PF03777”, was used to identify chaplins. Proteins possessing the Pfam domain, “PF01648”, with the E-values of < 10^–3^ were considered PPTases^107^. Additional analyses were conducted for the following proteins and metabolic pathway.

#### CAZyme clustering

CAZyme families of which at least 70% of the members were conserved in between 5 and 55% of the strains were collected. Orthologous groups conserved in at least 5% of the strains from these families were used to calculate the presence-absence matrix. UMAP was used for dimension reduction of the presence-absence matrix^45^. Clusters were identified by using HDBSCAN^44^.

#### Transcription factors and Sigma factor

Proteins predicted to possess the Sigma70_r2 (PF04542) domain and either Sigma70_r4 (PF04545) or Sigma70_r4_2 (PF08281) domain were considered σ^70^-family sigma factors. Sigma factors possessing both the Sigma70_r1_2 (PF00140) domain and the Sigma70_r3 (PF04539) domain were assigned to “group 1 or 2” and those possessing the Sigma70_r3 domain but not the Sigma70_r1_2 domain were assigned to “group 3”. All other sigma factors were assigned to “group 4”. Similarly, Pfam domains Sigma54_AID (PF00309), Sigma54_CBD (PF04963) and Sigma54_DBD (PF04552) were used to identify σ^54^-family sigma factors. A protein was predicted to be a transcription factors if it possessed one of the domain architectures used by P2TF^81^.

#### β-Ketoadipate pathway

KofamKOALA v1.3.0 was used to assign KO terms to proteins using the criteria used above^103^. Additionally, the proteins that had no KO terms assigned were still assigned highest score KO terms when independent E-value was < 10^–80^.

## Data and code availability

The pangenome data and code to process the data are available at the Secondary Metabolism Collaboratory (https://smc.jgi.lbl.gov/projects/) with no restriction to use.

## Supplementary Information

Supplementary Information 1.

Supplementary Information 2.
