# Supplementary material for: Comparative and pangenomic analysis of the genus Streptomyces
Source: Sci Rep. 2022 Nov 7;12:18909. doi: 10.1038/s41598-022-21731-1 (PMC9640686; doi:10.1038/s41598-022-21731-1)
Supplement: Supplementary file 1 — Supplementary Information 1. [file 41598_2022_21731_MOESM1_ESM.pdf]

Supplementary information

Comparative and pangenomic analysis of the genus *Streptomyces*

Hiroshi Otani[1, 2,] *, Daniel Udwary[1, 2], Nigel J. Mouncey[1, 2,] *

[1]DOE Joint Genome Institute and [2]Environmental Genomics and Systems Biology Division, Lawrence Berkeley National Laboratory, Berkeley, CA, 94720 USA

*To whom correspondence should be addressed. hotani@lbl.gov, nmouncey@lbl.gov

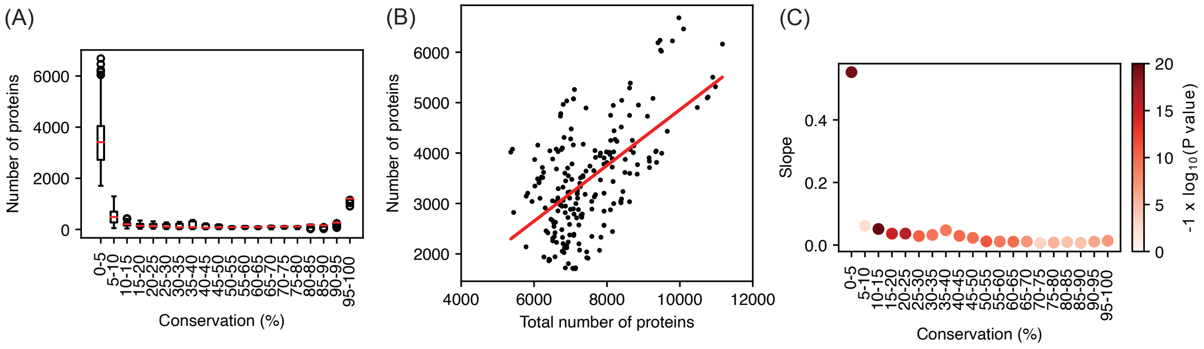

Figure S1. (A) Number of proteins in each bin of conservation. (B) Correlation between the total number of proteins and the number of proteins conserved in 0-5% strains encoded in the same genome. X axis is the total number of proteins each genome encodes and the y axis is the number of proteins conserved in 0-5% strains. Red line is the linear regression curve. (C) Correlation between the total number of proteins and the number of proteins in each bin of conservation. Y axis is the slopes of the regression curves. Dots are coloured based on the P values for their slopes.

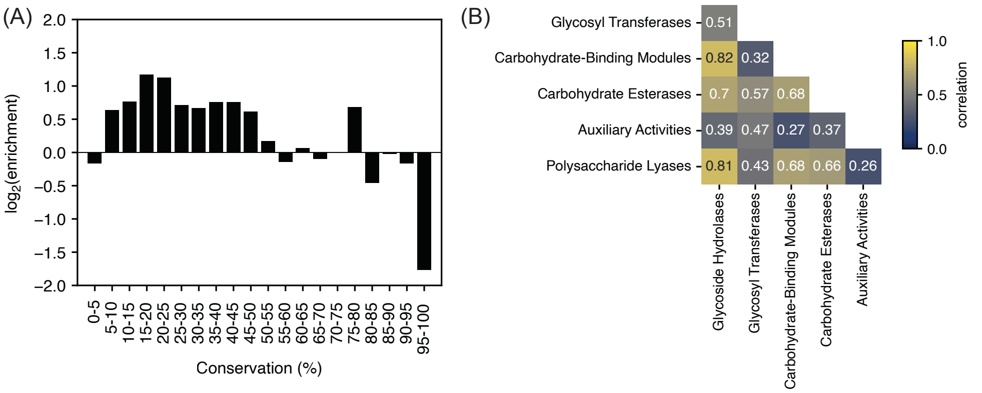

Figure S2. (A) Enrichment of CAZymes. Enrichment is the ratio between the number of CAZymes in each bin divided by the total number of CAZymes and the number of total proteins in each bin divided by the number of total proteins. (B) Pearson correlation coefficients between CAZyme groups.

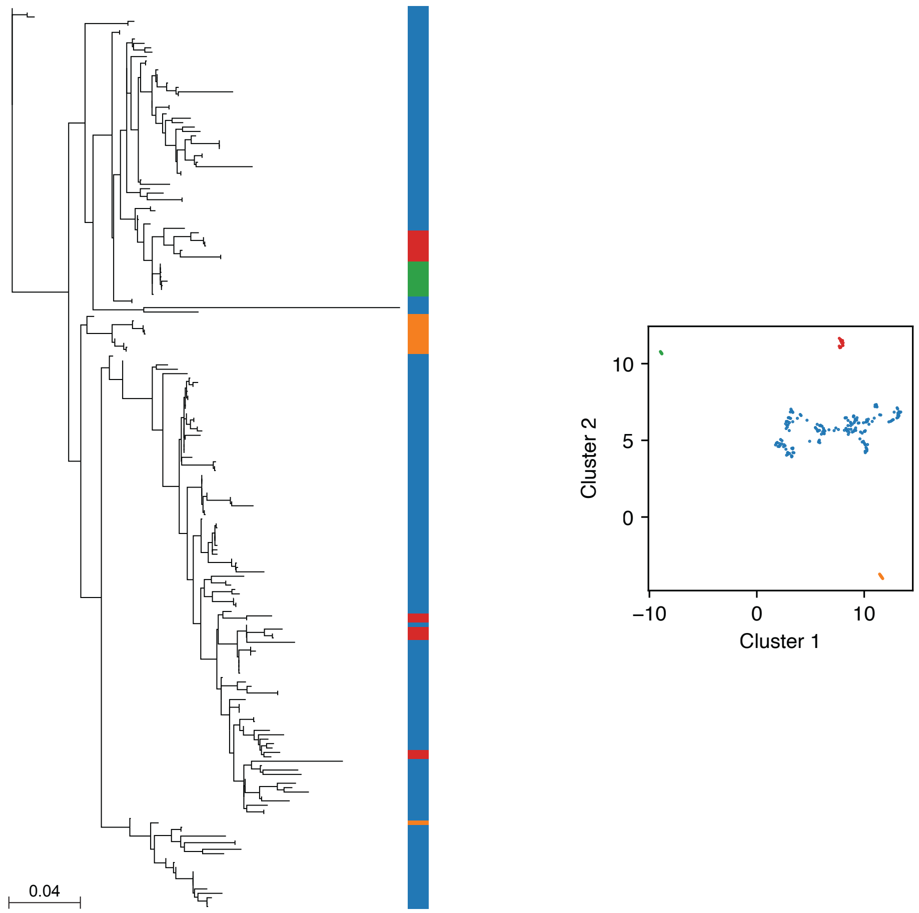

Figure S3. (A) Clustering the 205 genomes based on the presence or absence of each orthologous group from the 25 CAZyme families, and two-dimensional representation of the clusters by UMAP. The phylogenetic tree and the colour map of the UMAP classification are reproduced from Fig. 1.

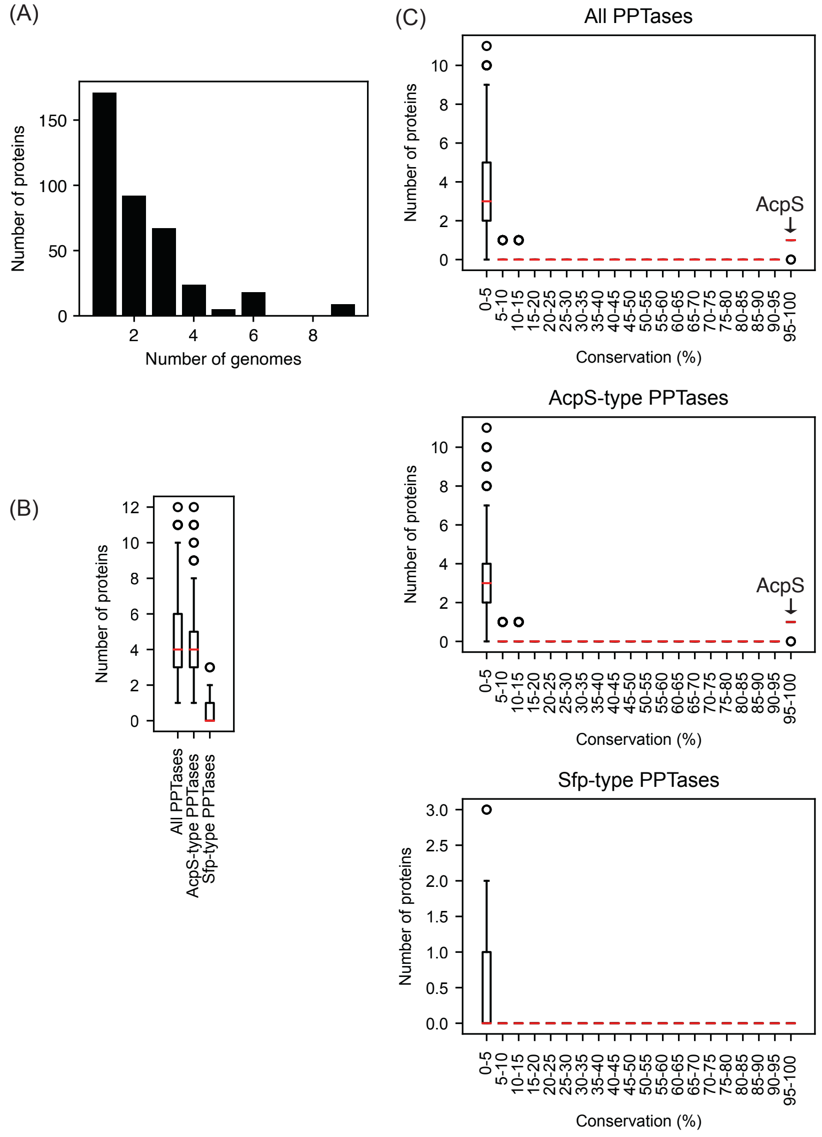

Figure S4. (A) Number of core enzymes for gamma-butyrolactone biosynthesis conserved in 1-9 strains. (B) Numbers of all, AcpS-type and Sfp-type PPTases in each genome. (C) Number of all PPTases in each bin in each genome.

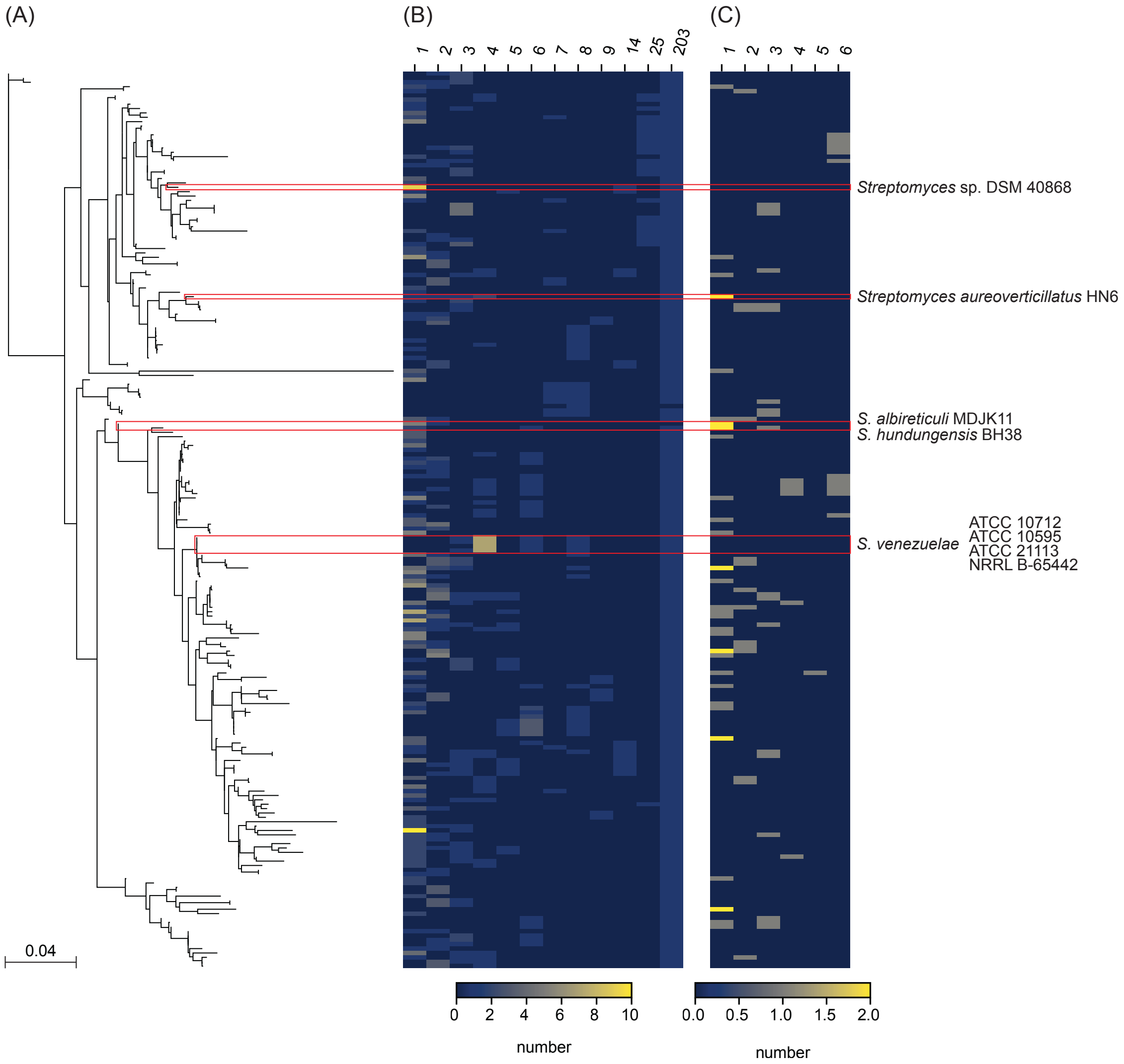

Figure S5. (A) Phylogenetic tree of the 205 streptomycetes using the 16S rRNA sequences. Sequences were aligned using PhyML. This figure is replication of Figure 1A. (B) The number of AcpS-type PPTases conserved in 1, 2, 3, 4, 5, 6, 7, 8, 9, 14, 25 and 203 strain(s). (C) The number of Sfp-type PPTases conserved in 1, 2, 3, 4, 5 and 6 strain(s).

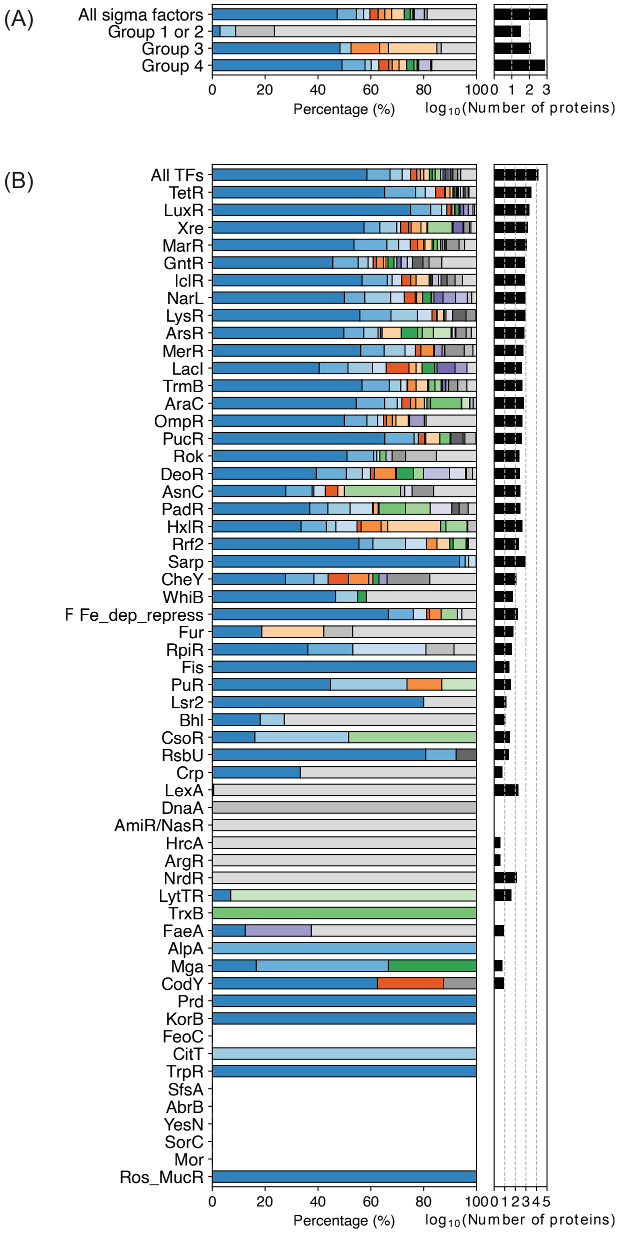

Figure S6. Distribution of conservation of sigma factors (A) and other transcription factor families (B) encoded inside secondary metabolite BGCs. For colour legends, see **Figure 3**.

Table S1. *Streptomyces* genomes used in this study.

Table S2. The numbers of CAZymes and CAZyme groups that each genome encoded.

Table S3. The numbers of the BGCs and core enzymes encoded in each genome.

Table S4. The number of each BGC type encoded in each genome.

Table S5. Sigma factors conserved in at least 95% strains.

Table S6. Other transcription factors conserved in at least 95% strains.
